# A 30-year update of the climbers and vascular epiphytes inventory of the Cerro Ñielol Natural Monument (La Araucanía, Chile): a database

**DOI:** 10.3897/BDJ.9.e72521

**Published:** 2021-09-15

**Authors:** Jimmy Pincheira-Ulbrich, Bárbara Vallejos, Jorge Huincaguelo, Ulises Zambrano, Fernando Peña-Cortés

**Affiliations:** 1 Laboratorio de Planificación Territorial, Departamento de Ciencias Ambientales, Facultad de Recursos Naturales, Universidad Católica de Temuco, Temuco, Chile Laboratorio de Planificación Territorial, Departamento de Ciencias Ambientales, Facultad de Recursos Naturales, Universidad Católica de Temuco Temuco Chile; 2 Pedagogía Media en Ciencias Naturales y Biología, Facultad de Educación, Universidad Católica de Temuco, Temuco, Chile Pedagogía Media en Ciencias Naturales y Biología, Facultad de Educación, Universidad Católica de Temuco Temuco Chile; 3 Geografía, Facultad de Recursos Naturales, Universidad Católica de Temuco, Temuco, Chile Geografía, Facultad de Recursos Naturales, Universidad Católica de Temuco Temuco Chile

**Keywords:** biodiversity, filmy ferns, forest, Mediterranean vegetation, phorophyte, vascular plants

## Abstract

**Background:**

Plant species diversity may be seriously threatened in ecotone zones under global climate change. Therefore, keeping updated inventories of indicator species seems to be a good strategy for monitoring wild areas located in these strips. The database comes from an inventory of climbers and vascular epiphytes conducted in the Cerro Ñielol Natural Monument, a small protected area (89 hectares) located in Chile's Mediterranean-temperate phytogeographic region, within the boundaries of the city of Temuco, La Araucaína Region.

The data represent the update of the first inventory carried out between 1980 and 1984. In this current contribution, data collection was carried out in 27 quadrats using the trails as transects. The data provide the record of 45 species (16 climbers, 15 epiphytes and 10 trees), including two accidental epiphytes (*Acerpsudoplatanus* L. and *Gavileaodoratissima* (L.) Endl. ex Griseb.), two species that can be found as epiphytes or terricolous (*Hymenophyllumtunbrigense* (L.) Sm. and *Nerteragranadensis* (Mutis ex L.f.) Druce) and one species (*Chusqueaquila* Kunth) that can be found as terricolous and climber. Species of interest were recorded on live trees (n = 51), snags (n = 9), stumps (n = 4), fallen log (n = 5) and on the forest soil (n = 17).

The most abundant climbers were *Hydrangeaserratifolia* (Hook. & Arn.) F. Phil. (n = 77 stems), *Lapageria* rosea Ruiz & Pav. (n = 70 stems), *Raukauavaldiviensis* (Gay) Frodin (n = 48 stems) and *Cissusstriata* Ruiz & Pav. (n = 33 stems). In contrast, the most abundant epiphytes were *Hymenophyllumplicatum* Kaulf. (n = 1728 fronds) and *Hymenophyllumtunbrigense* (L.) Sm. (n = 2375 fronds). These latter two species represent the highest frequency and abundance in the whole inventory, respectively. Several ecosystem traits are, in fact, new reports since the first inventory was conducted in 1980-1984; for example, the presence of the filmy fern *Hymenophyllumtunbrigense*, the record of the climber *Elytropuschilensis* , fallen logs or the species-host relationship. Accordingly, the database is made available in this manuscript.

**New information:**

This study updates the climbers and vascular epiphyte species list in the Cerro Ñielol Natural Monument, a small patch of forest under severe anthropogenic pressure. This protected area is characterised by floristic elements of the Mediterranean and temperate phytogeographic region of Chile, in a zone where forests have been severely deforested. The database includes the record of 45 species – including six species that were not recorded in the first inventory – in 211 records.

The main novelty of this contribution is the systematic classification of species, on ten traits rarely reported in a floristic inventory: (i) species taxonomic identity (as usual), (ii) species abundance (number of stems and fronds), (iii) habit (herb, shrub, subshrub, tree), (iv) growth form (accidental epiphyte, epiphyte, vine, liana, terricolous), (v) climbing mechanism (tendrils, adhesive roots, twining, scrambling), (vi) microhabitat (fallen log, footpath slope, soil, stump, trunk), (vii) host species (where appropriate), (viii) host condition (live, woody debris, snag), (ix) host diameter at breast height (DBH) and (x) target species found over 2.3 m on trees.

Thirty years after the first inventory conducted between 1980 and 1984, the climber assemblage has remained relatively stable over time, although there are some differences in species composition. Specifically, the climber *Elytropuschilensis* are recorded in the current inventory, but the *Mitrariacoccinea* (recorded in the first inventory) is not present. On the other hand, the epiphyte assemblage showed an increase in the species richness of filmy ferns, with five previously unrecorded species: *Hymenophyllumcuneatum*, *H.dicranotrichum*, *H.pectinatum*, *H.peltatum* and *H.tunbrigense*. One of the novel features was the presence of *Sarmientascandens* and *Synammiafeuillei* on a *Pinusradiata* D. Don tree. Additionally, the introduced species *Acerpseudoplatanus* is included, which is new to the Chilean vascular plant catalogue. All these data are available in the present manuscript.

## Introduction

Species inhabiting small patches of forest are under strong pressure, especially when these patches are isolated and immersed in an anthropogenic matrix. A case in point is the wilderness areas located in the Mediterranean-temperate phytogeographic interaction zone in central Chile (Myers et al. 2000). The problem with small patches is that they also maintain relatively small populations of most species (Zotz and Bader 2009, Campbell et al. 2014, Haddad et al. 2015), which could disappear due to events, such as (Shaffer 1981): (i) demographic stochasticity (e.g. variability of reproductive success), (ii) environmental stochasticity (e.g. changes in light levels in the habitat), (iii) natural catastrophes (e.g. fires) and (iv) reduced genetic diversity (loss of alleles). The physical and biological effects that the matrix exerts on these small wilderness areas may be exacerbated under global climate change, seriously limiting the ability of these areas to maintain their biodiversity levels (Mantyka-pringle et al. 2011). The IPCC (Hoegh-Guldberg et al. 2018) predicts with medium confidence that 8% of plant species will become extinct due to a reduction in half of their geographic range, determined by a 1.5°C climate warming. In Chile, the effect of climate change is expected to allow sclerophyll forest to move southwards over the region currently occupied by temperate forest (Pliscoff et al. 2012). Indeed, the Mediterranean-type climate of central Chile is projected to expand by 129-153% of its current size by the end of the 21st century (Klausmeyer and Shaw 2009).

The ecotonal band between the Mediterranean-type and temperate phytogeographical regions may present one of the most significant challenges for species conservation, as they are generally restricted in extent and are characterised by rapid environmental and biological change (Kark 2012). Transition zones are crucial for the functioning of ecosystems. They possess high diversity, endemism and unique genotypes, mainly because they function as refuges for rare or sensitive species to environmental change. These latter species would have an essential role as indicators of climate change (Klausmeyer and Shaw 2009, Martay et al. 2016). One of the groups of plants sensitive to these changes are climbers and vascular epiphytes, which depend on forest trees for survival and show differentiated responses to environmental gradients so that they can become indicator species for environmental and biological changes in the ecosystem (van der Heijden and Phillips 2008, Pincheira-Ulbrich et al. 2018). Indicator plants seem to be a reasonable starting point for a long-term monitoring programme since changes in the diversity of these species are amongst the best available predictors of the diversity for other taxa (Pereira and Cooper 2006).

**Figure 1.**
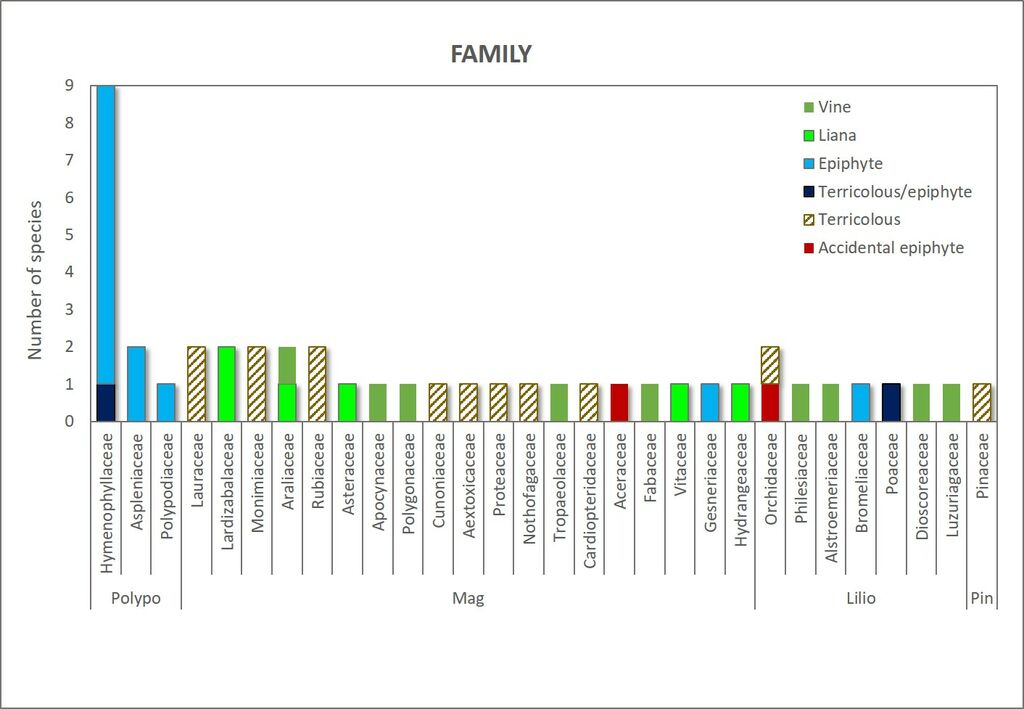
Species richness classified by family, growth form and phylum. Polypo= Polypodiopsida, Mag = Magnoliophyta, Lilio = Liliopsida, Pin = Pinophyta.

**Figure 2.**
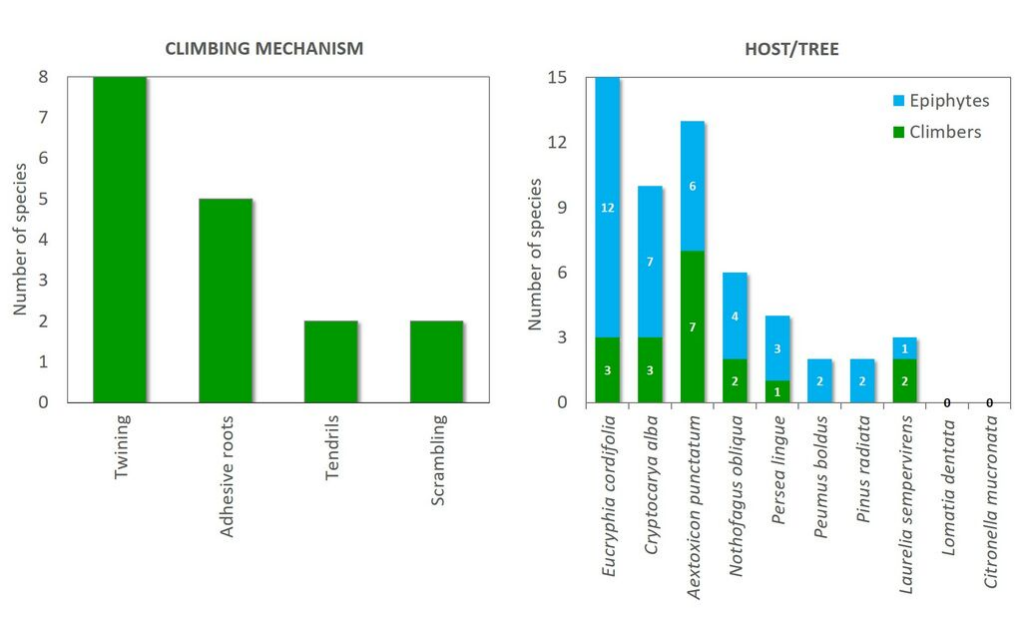
Climbing mechanisms and host trees for climbers and epiphytes.

This contribution updates the inventory of climbers and vascular epiphytes carried out in the Cerro Ñielol Natural Monument between 1980 and 1984 (Hauenstein et al. 1988). This small protected wilderness area (89 hectares) is located within the urban limit of the city of Temuco in the ecotonal fringe between the Mediterranean-type and temperate phytogeographic regions of Chile (Table 1, Suppl. material 1). Data describe (i) species taxonomic identity (Fig. 1), (ii) species abundance (number of stems and fronds), (iii) habit (herb, shrub, subshrub, tree [Table 1]), (iv) growth form (accidental epiphyte [Fig. 5], epiphyte [Fig. 4], vine [Fig. 3], liana [Fig. 6], terricolous), (v) climbing mechanism (tendrils, adhesive roots, twining, scrambling [Fig. 2]), (vi) microhabitat (fallen log, footpath slope, soil, stump, trunk), (vii) host species (where appropriate [Fig. 2]), (viii) host condition (live, woody debris, snag), (ix) host diameter at breast height (DBH) and (x) target species found over 2.3 m on trees. Several of the ecosystem features are, in fact, new reports since the first inventory was conducted, for example, the presence of the filmy fern *Hymenophyllumtunbrigense*, the record of the climber *Elytropuschilensis*, the fallen logs or the species-host relationship.

## General description

### Purpose

This contribution provides baseline information for the monitoring of climbing plants and vascular epiphytes, species that are potential indicators of environmental and habitat structure changes. The geographical location of the sampling quadrats facilitates this work. The data are expected to contribute to the local assessment and conservation of species in this protected wilderness area which is subject to strong anthropogenic pressure.

## Project description

### Study area description

The Cerro Ñielol Natural Monument is located on the southern boundary of the mountain range "Huimpil-Ñielol" (38°43' South Latitude and 72°35 West Longitude; Fig. 8). The area extends into the Intermediate Depression of the Araucanía Region in Chile. To the north, it is bordered by agroforestry owners and Mapuche (indigenous) communities, while, to the south-east and south-west, it is within the urban radius of the city of Temuco. The climate is temperate-humid with Mediterranean-type influence, average annual rainfall is 1,325 mm, with no rain in January and February. The average annual temperature is 12°C, while the average maximum in the hottest month is 25.3°C and the average minimum temperature is 4.1°C (Luebert and Pliscoff 2006). The forest is composed of temperate forest species, such as *Nothofagusobliqua* and *Eucryphiacordifolia* and sclerophyllous forest species, dominated by *Cryptocaryaalba* (Hauenstein et al. 1988).

### Design description

**Figure 7.**
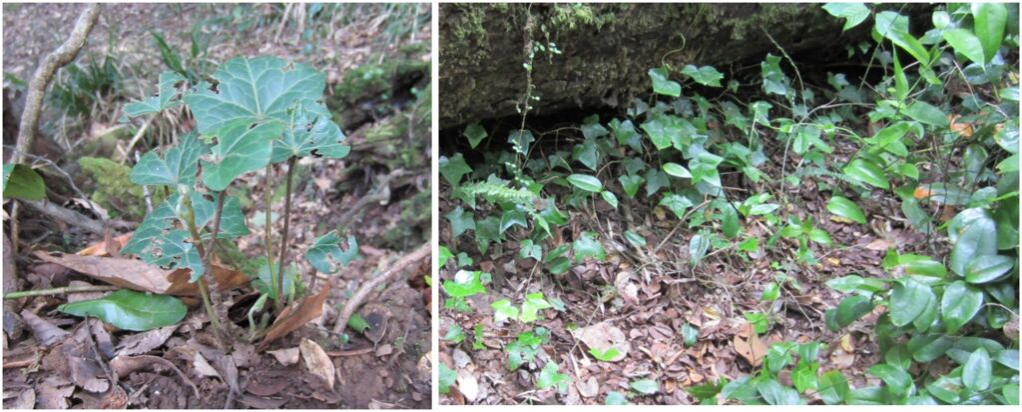
*Hederahelix* (Araliaceae), introduced species.

**Figure 8.**
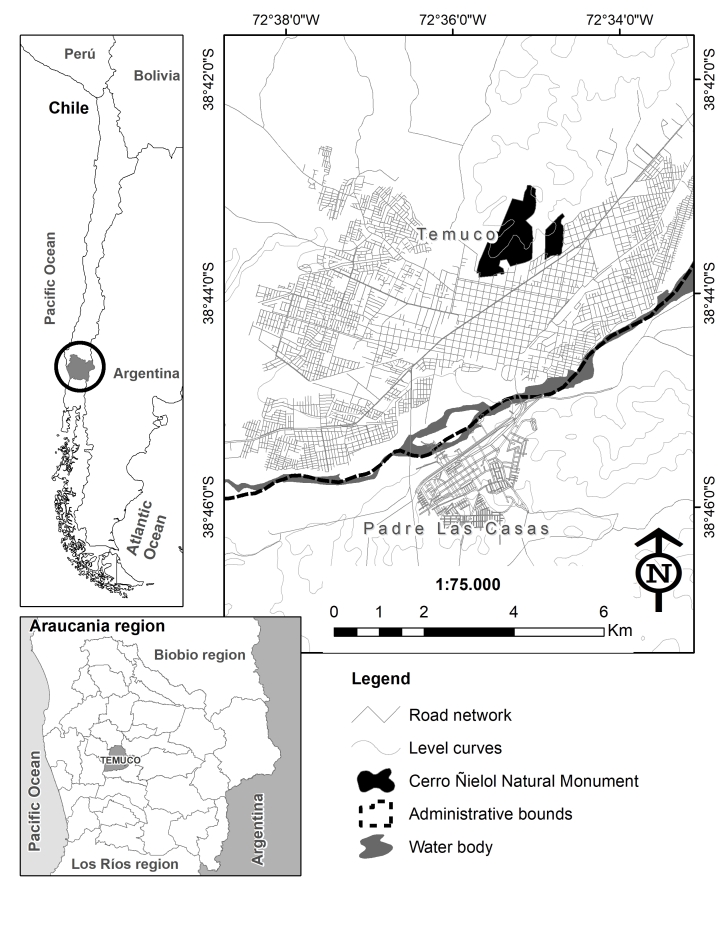
Location of the Cerro Ñielol Natural Monument.

The sampling design was non-random in the hope of including as much variation in microhabitats and rare species as possible (Diekmann et al. 2007, Croft and Chow-Fraser 2009). Vascular epiphytes, trees, shrubs and both woody (lianas) and non-woody vines were recorded (both native and introduced species; e.g. Fig. 7). Data collection was carried out between November 2014 and June 2015 and required 13 effective days in the field. Twenty-seven circular quadrats of three metres in diameter (7.06 m^2^) were established, maintaining a distance of at least 10 metres between quadrats (e.g. Pincheira-Ulbrich et al. 2016). Species sampling followed an observational protocol from the base of the ground to 2.3 m above the trunk (Flores-Palacios and García-Franco 2001). Regular observations were made above 2.3 m in search of new species records. The quadrats were arranged on trails that were used as transects (Brower et al. 1990).

## Sampling methods

### Sampling description

Data collection was carried out between 2014 and 2015 and required 13 effective days in the field. Sampling followed a transect sampling observations protocol (Brower et al. 1990), following footpaths to select sampling points to enter the forest. Field notes and photographs taken along the transect were reviewed in the laboratory. Ten types of data were described: (i) taxonomic identity, following Marticorena and Rodríguez (Rodríguez 1995, Marticorena and Rodríguez 2001, Marticorena and Rodríguez 2003, Marticorena and Rodríguez 2005, Marticorena and Rodríguez 2011), (ii) species abundance (number of stems and fronds, e.g. Pincheira-Ulbrich et al. 2016), (iii) habit (herb, shrub, subshrub, tree) according to Rodriguez et al. (2018), (iv) growth form (accidental epiphyte, epiphyte, climber, liana, terricolous), according to Marticorena et al. (2010) and Rodríguez et al. (2009), (v) climbing mechanism (tendrils, adhesive roots, twining, scrambling), according to Sperotto et al. (2020), (vi) microhabitat (fallen log, footpath slope, soil, stump, trunk) as observed in the field, (vii) host species (where appropriate), (viii) host condition (live, woody debris, snag), (ix) host diameter at breast height (DBH) and (x) target species found over 2.3 m on trees. Taxonomic nomenclature followed Rodriguez et al. (2018) and the International Plant Name Index (IPNI 2021). Species recorded in the first inventory are included. This was conducted using the Braun-Blanquet phytosociological method, in which 15 forest censuses of 400 m² were defined in the forest (Hauenstein et al. 1988).

## Geographic coverage

### Description

The Cerro Ñielol Natural Monument is located on the southern boundary of the mountain range "Huimpil-Ñielol", which extends into the Intermediate Depression of the Araucanía Region in Chile.

### Coordinates

38°43'42'' and 38°43'02'' Latitude; 72°34'42'' and 72°35'41'' Longitude.

## Traits coverage

Climbing plants, vascular epiphytes, trees and shrubs

## Temporal coverage

**Data range:** 2014-11-06 – 2015-6-26.

## Usage licence

### Usage licence

Creative Commons Public Domain Waiver (CC-Zero)

## Data resources

### Data package title

EpiphytevinesDataset

### Number of data sets

1

### Data set 1.

#### Data set name

A 30-year update of the climbers and vascular epiphyte inventory of the Cerro Ñielol Natural Monument: a database

#### Data format

csv

#### Number of columns

19

#### Data format version

csv

#### Description

The dataset provides the record of 45 species (16 climbers, 15 epiphytes and 10 trees) including two accidental epiphytes (*Acerpsudoplatanus* and *Gavileaodoratissima*), two species that can be found as epiphytes or terricolous (*Hymenophyllumtunbrigense* and *Nerteragranadensis*) and one species (*Chusqueaquila*) that can be found as terricolous and climber. Species of interest were recorded on live trees (n = 51), snags (n = 9), stumps (n = 4), fallen log (n = 5) and on the forest soil (n = 17) in 211 records. Several of the biological backgrounds presented here have not been reported in literature.

**Data set 1.** 

Column label	Column description
Id	Row identifier
Quadrant	Sampling quadrant number
Latitude	Geographic coordinate that specifies the north–south position of a point on the Earth's surface
Longitude	Geographic coordinate that specifies the east–west position of a point on the Earth's surface
ID species	Record number of climbers and vascular epiphytes species. NA = Not applicable
Species	Scientific name of climbers and vascular epiphytes species. NA = Not applicable
Abundance	Abundance of climbers (number of stems) and vascular epiphytes (number of fronds). NA = Not applicable, UD = Undefined
Habit	Growth habit according to literature. Herb, shrub, subshrub, tree. NA = Not applicable
Growth form	Growth form according to literature. Accidental epiphyte, epiphyte, climber, liana, terricolous. NA = Not applicable
Climbing mechanism	climbing mechanism of climbers. Tendrils, adhesive roots, twining, scrambling. NA = Not observed in the field
ID Host/substrate	Record number of host or substrate. Also includes trees without species occurrence
Microhabitat	Microhabitat where species grow. Fallen log, footpath slope, soil, stump, trunk. NA = Not applicable
Host/tree	Scientific name of host and non-species trees. NA = Not applicable, UD = Undefined
Host condition	Living trees and tree debris. Live, woody debris, snag. NA = Not applicable
Host DHB	Host diameter at breast height. NA = Not applicable, UD = Undefined
2.3 m in height	Species found above 2.3 m in the trunk
Date of data collection	Date
Observer name	Name of the person who collected data in the field
Notes	Other species observed inside or outside the quadrant

## Additional information

The results showed an increase in the number of species of Hymenophyllum (film ferns). We suggest that this can be explained by at least three situations that need to be further investigated:

1. The change in microhabitat conditions driven by forest dynamics, which would explain the presence of these species today.
2. The misclassification of Hymenophyllum species in the first inventory, because there was limited access to manuals and taxonomic sources available when the species were identified, requiring specialists in the field.
3. The sampling design of the first inventory which is not directly comparable to the current study.

## Supplementary Material

751E14BE-8C6E-55CC-8E36-12DC843D185B 10.3897/BDJ.9.e72521.suppl1 Supplementary material 1 A 30-year update of the climbers and vascular epiphyte inventory of the Cerro Ñielol Natural Monument: a database Data type Abundance Brief description The dataset provides the record of 45 species (16 climbers, 15 epiphytes and 10 trees) including two accidental epiphytes (*Acerpsudoplatanus* and *Gavileaodoratissima*), two species that can be found as epiphytes or terricolous (*Hymenophyllumtunbrigense* and *Nerteragranadensis*) and one species (*Chusqueaquila*) that can be found as terricolous and climber. Species of interest were recorded on live trees (n = 51), snags (n = 9), stumps (n = 4), fallen log (n = 5) and on the forest soil (n = 17) in 211 records. File: oo_580953.csv https://binary.pensoft.net/file/580953 Jimmy Pincheira Ulbrich

## Figures and Tables

**Figure 3a.**
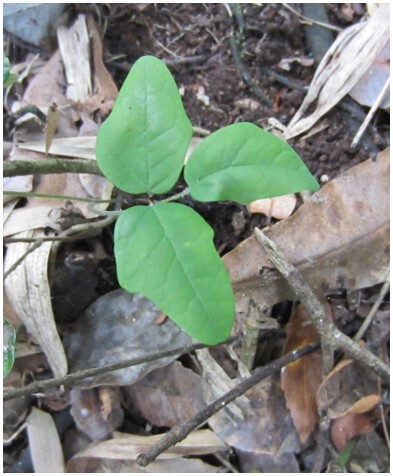
*Boquilatrifoliolata* (Lardizabalaceae)

**Figure 3b.**
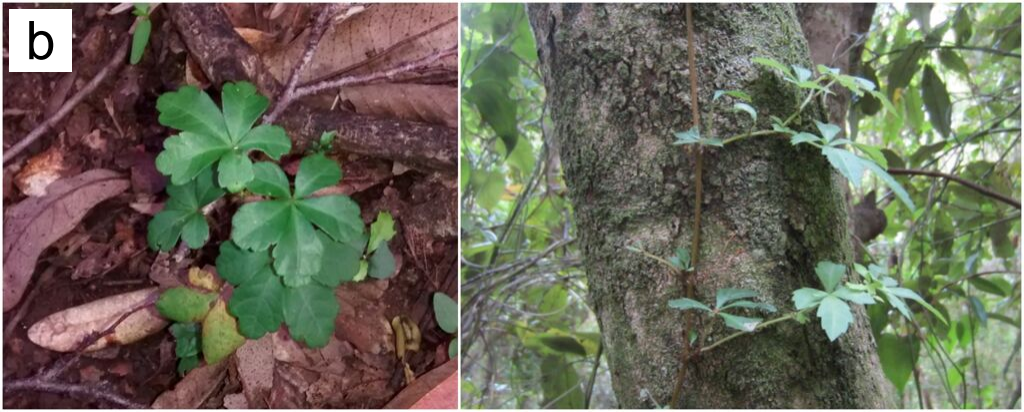
*Cissusstriata* (Vitaceae)

**Figure 3c.**
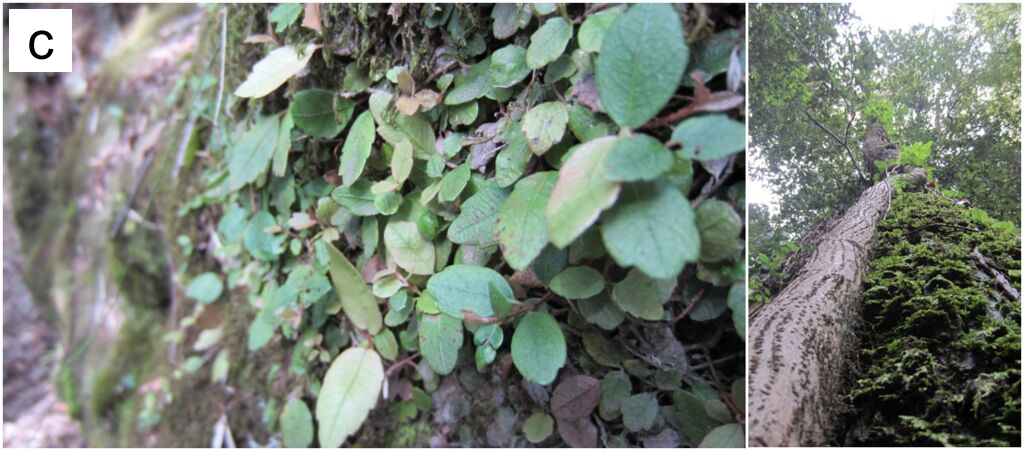
*Hydrangeaserratifolia* (Hydrangeaceae)

**Figure 3d.**
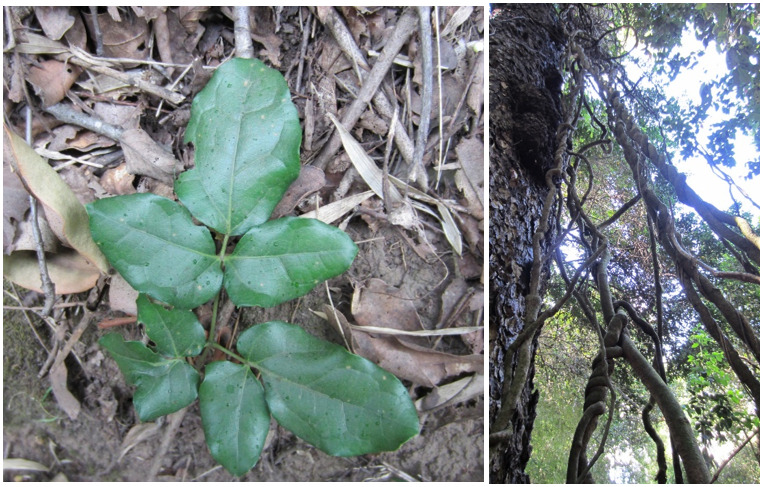
*Lardizabalabiternata* (Lardizabalaceae)

**Figure 3e.**
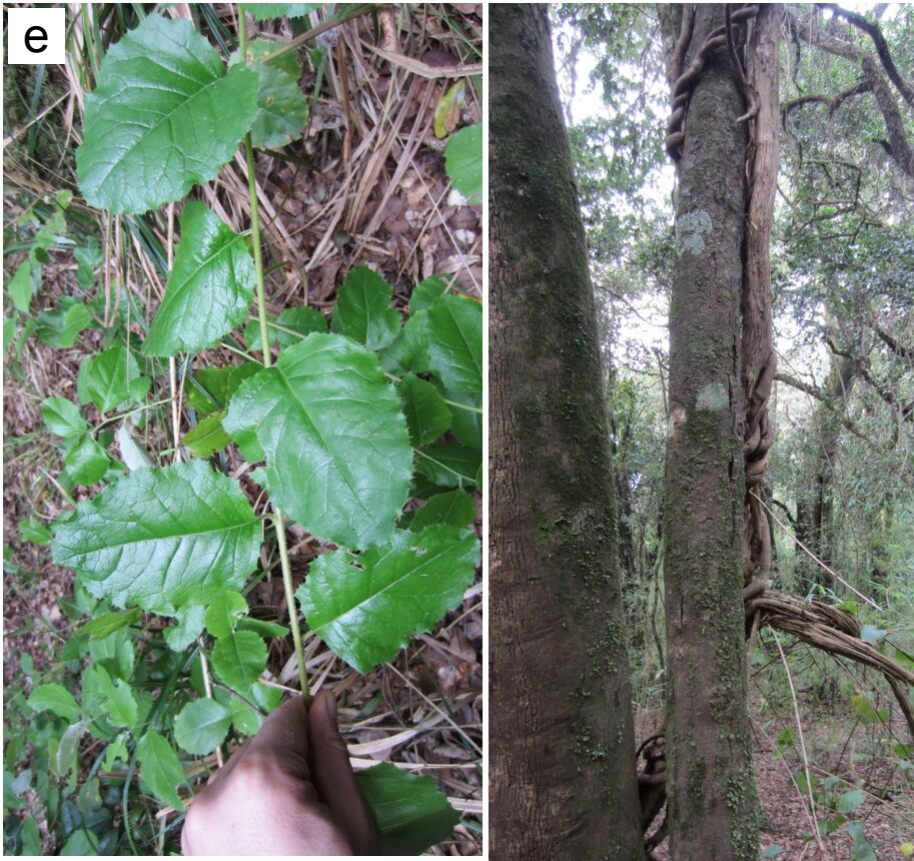
*Proustiapyrifolia* (Asteraceae)

**Figure 3f.**
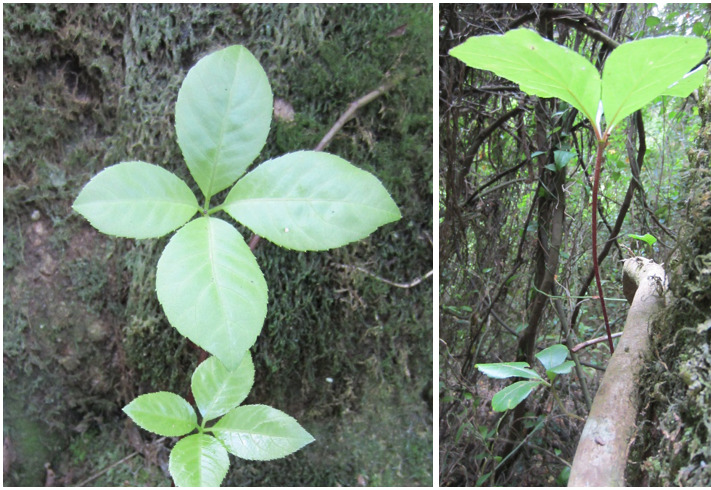
*Raukauavaldiviensis* (Araliaceae)

**Figure 4a.**
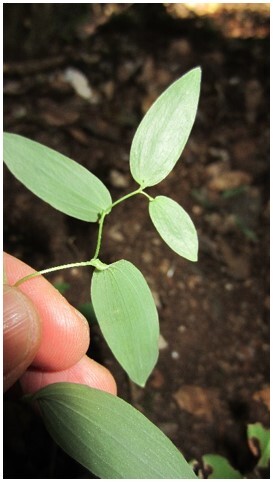
*Bomareasalsilla* (Alstroemeriaceae)

**Figure 4b.**
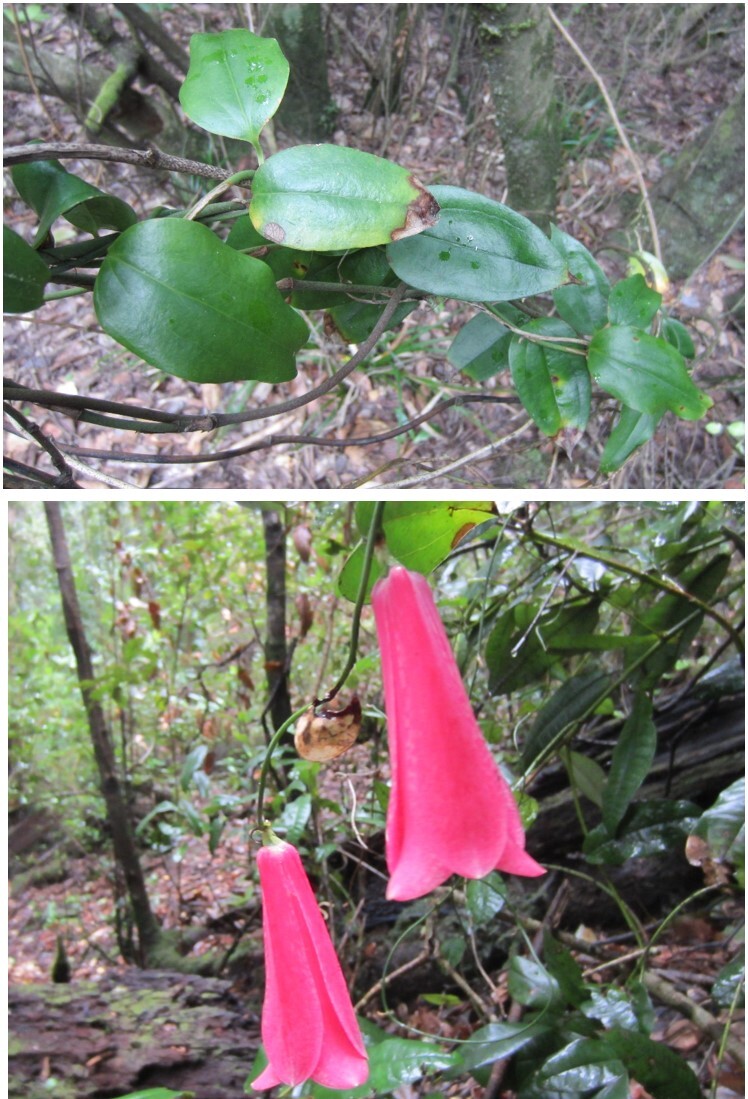
*Lapageriarosea* (Philesiaceae)

**Figure 4c.**
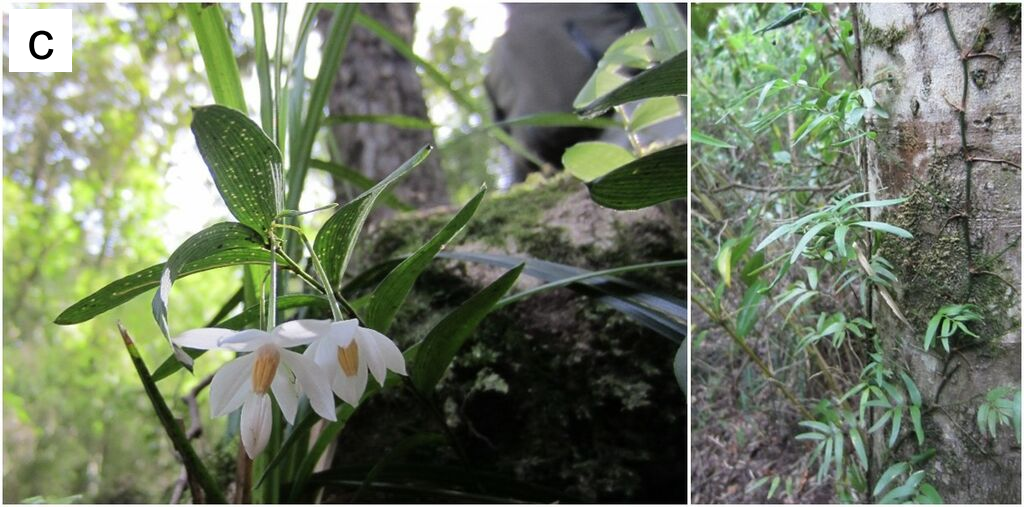
*Luzuriagaradicans* (Luzuriagaceae)

**Figure 4d.**
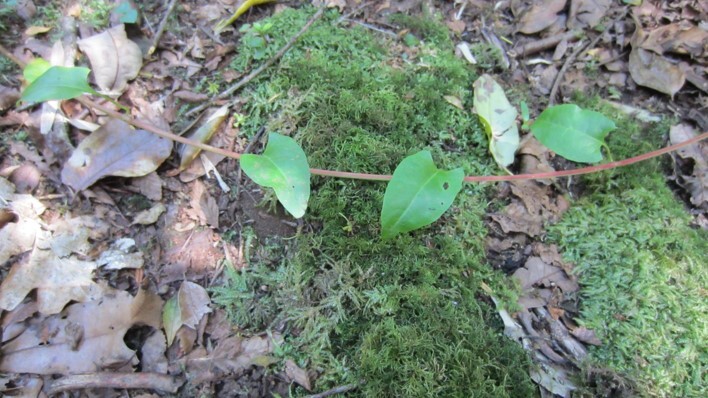
*Muehlenbeckiahastulata* (Polygonaceae)

**Figure 5a.**
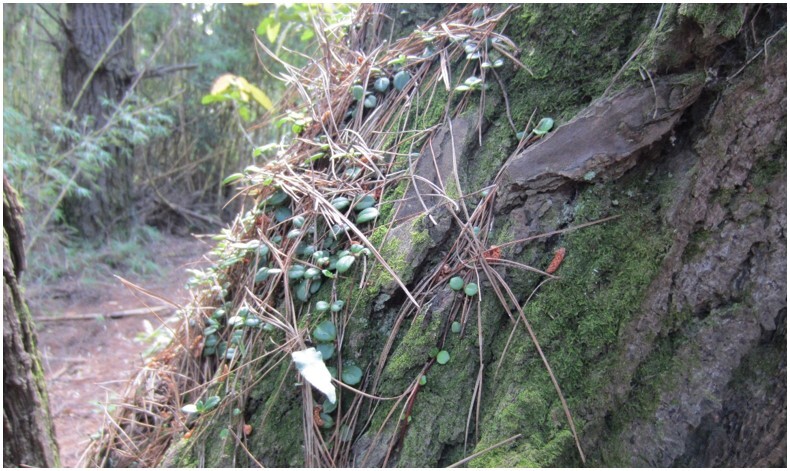
*Sarmientascandens* (Gesneriaceae) growing on *Pinusradiata* (Pinaceae)

**Figure 5b.**
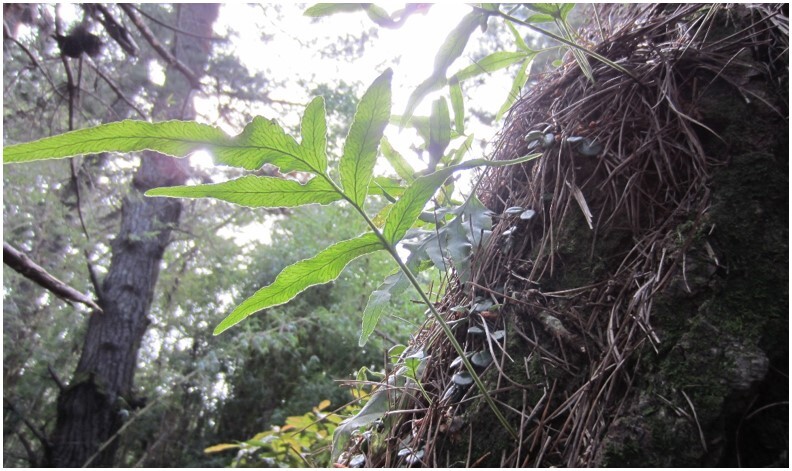
*Synammiafeuillei* (Polypodiaceae) growing on *Pinusradiata* (Pinaceae)

**Figure 5c.**
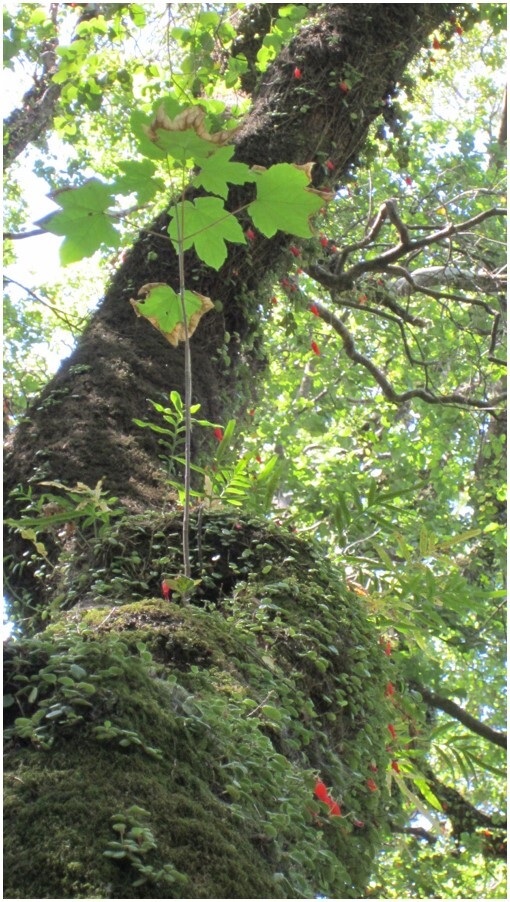
*Acerpseudoplatanus* (Aceraceae) growing on *Cryptocaryaalba* (Lauraceae)

**Figure 5d.**
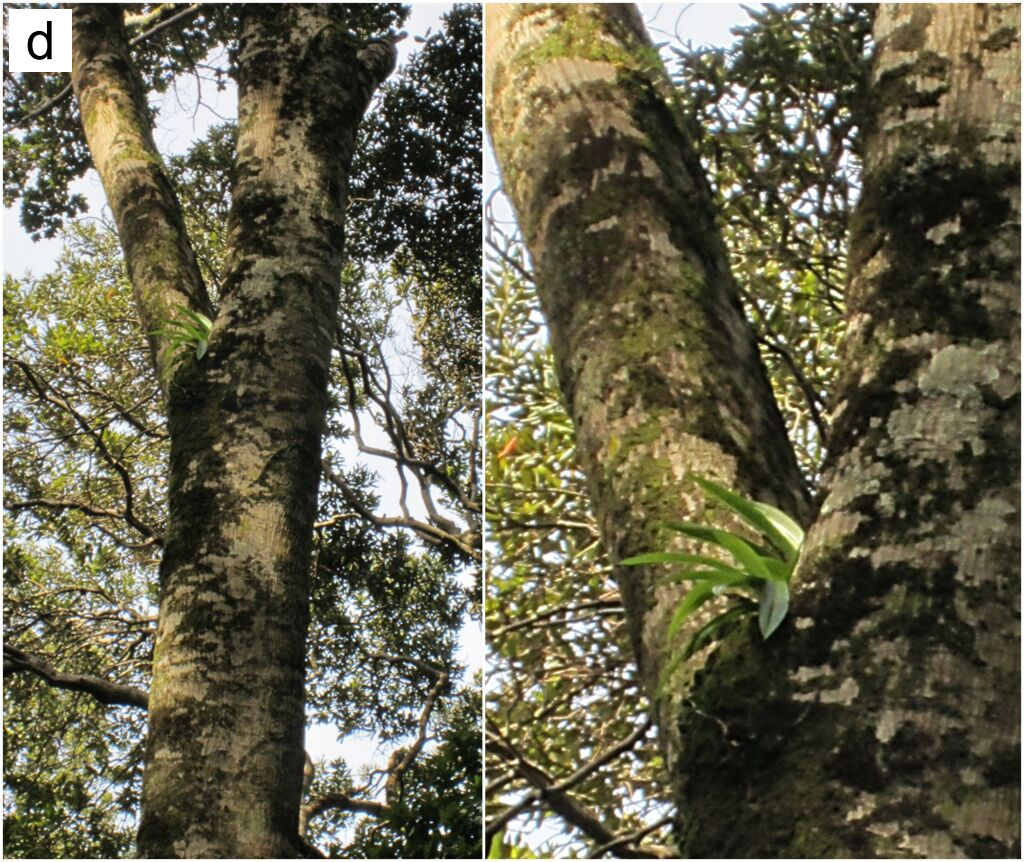
*Gavileaodoratissima* (Orchidaceae) growing on *Eucryphiacordifolia* (Cunoniaceae)

**Figure 6a.**
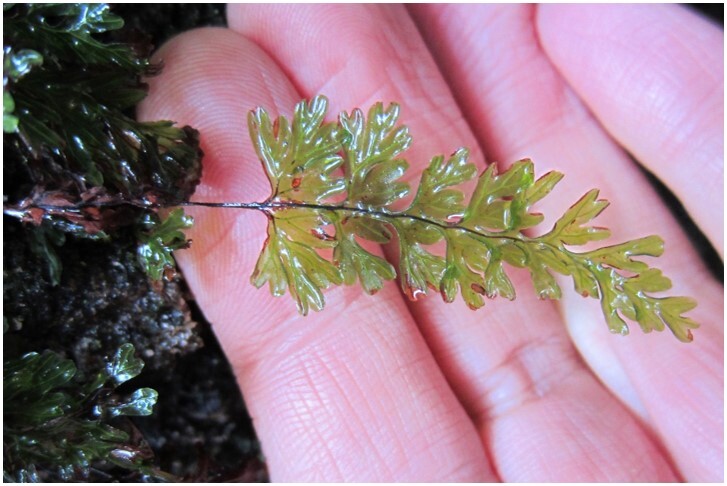
*Hymenophyllumcuneatum* (Hymenophyllaceae)

**Figure 6b.**
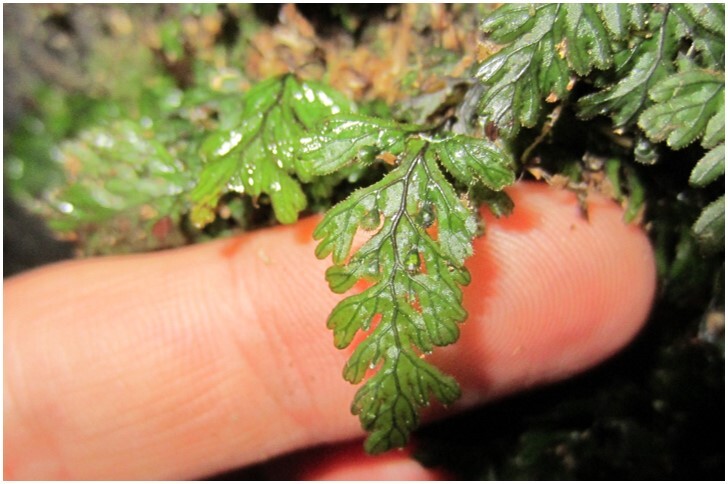
*Hymenophyllumdicranotrichum* (Hymenophyllaceae)

**Figure 6c.**
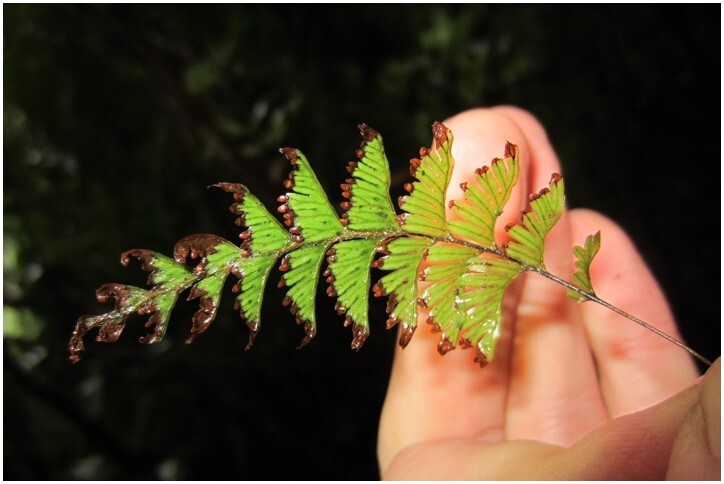
*Hymenophyllumpectinatum* (Hymenophyllaceae)

**Figure 6d.**
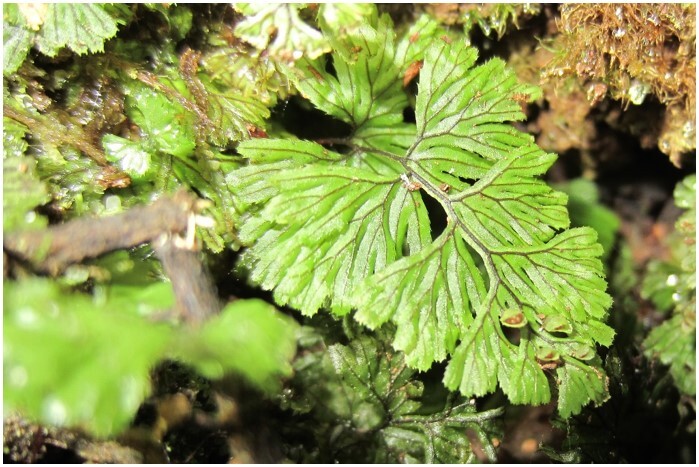
*Hymenophyllumtunbrigense* (Hymenophyllaceae)

**Figure 6e.**
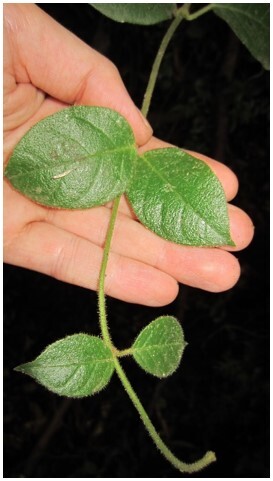
*Elytropuschilensis* (Apocynaceae)

**Figure 6f.**
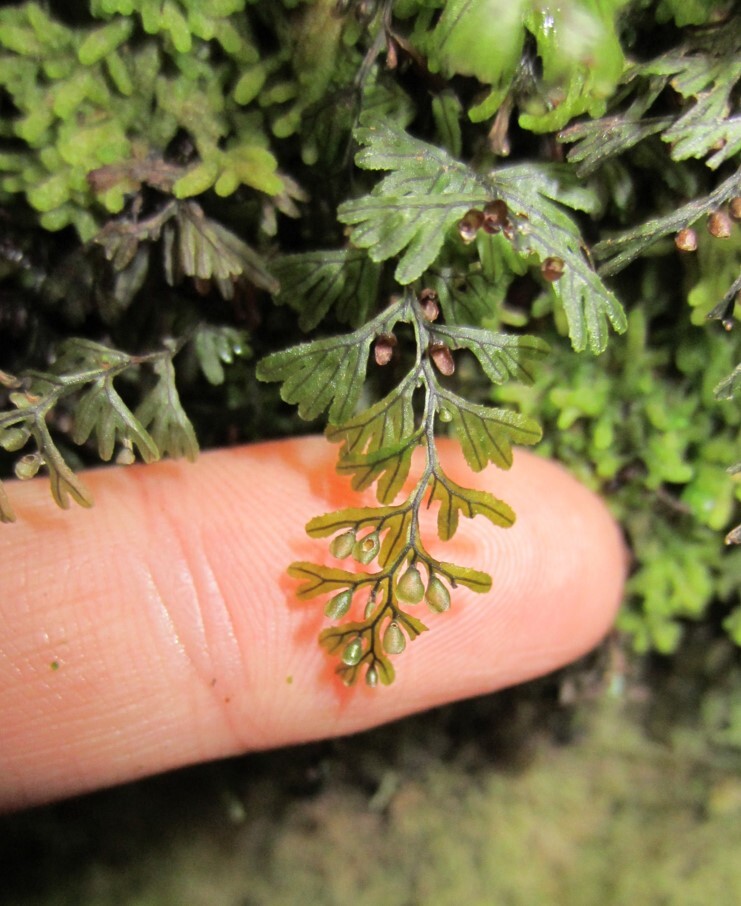
*Hymenophyllumpeltatum* (Hymenophyllaceae)

**Table 1.** Climbers, vascular epiphytes and trees of the Cerro Ñielol Natural Monument. Species Classification according to the criteria of Phyllum, family, growth form, habit and geographical origin are based on Rodriguez et al. (2018) and IPNI (2021). Classification of species in the Mediterranean-type and/or temperate phytogeographical regions follows Teneb et al. (2004), Marticorena et al. (2010) and Rodríguez et al. (2009). Native: Species are naturally distributed in Chile and other areas of South America. Endemic: Species only found in Chile. Phytogeographic region (PHYTO): TE = common species in temperate forest, ME = common species in the Mediterranean-type forest. Geographic origin (GEO): EN = endemic, IN = introduced, IN/na = introduced/naturalised, NA = native. * New species recorded. The Table includes the results of the first inventory conducted by Hauenstein et al. (1988).

**n**	**Specie**	**Phylum**	**Family**	**Growth form**	**Habit**	**PHYTO**	**GEO**	**First inventory**	**Current inventory**
1	*Acerpseudoplatanus* L.	Magnoliophyta	Aceraceae	Accidental epiphyte	Tree	TE - ME	IN/na	1	1
2	*Aextoxiconpunctatum* Ruiz & Pav.	Magnoliophyta	Aextoxicaceae	Terricolous	Tree	TE - ME	NA	1	1
3	*Aspleniumdareoides* Desv.	Polypodiopsida	Aspleniaceae	Epiphyte	Herb	TE - ME	NA	1	1
4	*Aspleniumtrilobum* Cav.	Polypodiopsida	Aspleniaceae	Epiphyte	Herb	TE	NA	1	1
5	*Bomareasalsilla* (L.) Herb	Liliopsida	Alstroemeriaceae	Vine	Herb	ME	NA	1	1
6	*Boquilatrifoliolata* (DC.) Decne.	Magnoliophyta	Lardizabalaceae	Liana	Shrub	TE - ME	NA	1	1
7	*Chusqueaquila* Kunth	Liliopsida	Poaceae	Vine/ terricolous	Herb	TE - ME	EN	1	1
8	*Cissusstriata* Ruiz & Pav.	Magnoliophyta	Vitaceae	Liana	Shrub	TE - ME	NA	1	1
9	*Citronellamucronata* (Ruiz & Pav.) D. Don	Magnoliophyta	Cardiopteridaceae	Terricolous	Tree	ME	EN	1	1
10	*Cryptocaryaalba* (Molina) Looser	Magnoliophyta	Lauraceae	Terricolous	Tree	ME	EN	1	1
11	*Dioscorea* spp.	Liliopsida	Dioscoreaceae	Vine	Herb	TE - ME	NA	1	1
12	*Dioscoreaauriculata* Poepp.	Liliopsida	Dioscoreaceae	Vine	Herb	TE - ME	NA	1	0
13	*Elytropuschilensis* (A. DC.) Müll. Arg.*	Magnoliophyta	Apocynaceae	Vine	Shrub	TE - ME	NA	0	1
14	*Eucryphiacordifolia* Cav.	Magnoliophyta	Cunoniaceae	Terricolous	Tree	TE	NA	1	1
15	*Fasciculariabicolor* (Ruiz & Pav.) Mez	Liliopsida	Bromeliaceae	Epiphyte	Herb	TE - ME	EN	1	1
16	*Galiumhypocarpium* (L.) Endl. ex Griseb.	Magnoliophyta	Rubiaceae	Terricolous	Herb	TE - ME	NA	1	1
17	*Gavileaodoratissima* Poepp.	Liliopsida	Orchidaceae	Accidental epiphyte	Herb	TE - ME	NA	1	1
18	*Gavilea* spp.	Liliopsida	Orchidaceae	Terricolous	Herb	TE - ME	NA	1	1
19	*Hederahelix* L.	Magnoliophyta	Araliaceae	Vine	Shrub	TE - ME	IN/na	1	1
20	*Hydrangeaserratifolia* (Hook. & Arn.) F. Phil.	Magnoliophyta	Hydrangeaceae	Liana	Shrub	TE - ME	NA	1	1
21	*Hymenophyllumcaudiculatum* Mart.	Polypodiopsida	Hymenophyllaceae	Epiphyte	Herb	TE	NA	1	1
22	*Hymenophyllumcuneatum* Kunze*	Polypodiopsida	Hymenophyllaceae	Epiphyte	Herb	TE	EN	0	1
23	*Hymenophyllumdentatum* Cav.	Polypodiopsida	Hymenophyllaceae	Epiphyte	Herb	TE	NA	1	1
24	*Hymenophyllumdicranotrichum* (C. Presl) Hook. exSadeb.*	Polypodiopsida	Hymenophyllaceae	Epiphyte	Herb	TE	EN	0	1
25	*Hymenophyllumkrauseanum* Phil.	Polypodiopsida	Hymenophyllaceae	Epiphyte	Herb	TE	NA	1	1
26	*Hymenophyllumpectinatum* Cav.*	Polypodiopsida	Hymenophyllaceae	Epiphyte	Herb	TE	NA	0	1
27	*Hymenophyllumpeltatum* (Poir.) Desv.*	Polypodiopsida	Hymenophyllaceae	Epiphyte	Herb	TE - ME	NA	0	1
28	*Hymenophyllumplicatum* Kaulf.	Polypodiopsida	Hymenophyllaceae	Epiphyte	Herb	TE	NA	1	1
29	*Hymenophyllumsecundum* Hook. & Grev.	Polypodiopsida	Hymenophyllaceae	Epiphyte	Herb	TE	NA	1	0
30	*Hymenophyllumtunbrigense* (L.) Sm.*	Polypodiopsida	Hymenophyllaceae	Terricolous/ Epiphyte	Herb	TE - ME	NA	0	1
32	*Lapageriarosea* Ruiz & Pav.	Liliopsida	Philesiaceae	Vine	Shrub	TE - ME	EN	1	1
33	*Lardizabalabiternata* Ruiz & Pav.	Magnoliophyta	Lardizabalaceae	Liana	Shrub	ME	EN	1	1
34	*Laureliasempervirens* (Ruiz & Pav.) Tul.	Magnoliophyta	Monimiaceae	Terricolous	Tree	TE	EN	1	1
35	*Lomatiadentata* (Ruiz & Pav.) R. Br.	Magnoliophyta	Proteaceae	Terricolous	Tree	TE - ME	NA	1	1
36	*Luzuriagaradicans* Ruiz & Pav.	Liliopsida	Luzuriagaceae	Vine	Subshrub	TE - ME	NA	1	1
37	*Mitrariacoccinea* Cav.	Magnoliophyta	Gesneriaceae	Vine	Herb	TE - ME	NA	1	0
38	*Muehlenbeckiahastulata* (Sm.) I.M. Johnst.	Magnoliophyta	Polygonaceae	Vine	Shrub	TE - ME	NA	1	1
39	*Nerteragranadensis* (Mutis ex L.f.) Druce	Magnoliophyta	Rubiaceae	Terricolous/ Epiphyte	Herb	TE - ME	NA	1	1
40	*Nothofagusobliqua* (Mirb.) Oerst.	Magnoliophyta	Nothofagaceae	Terricolous	Tree	TE	NA	1	1
41	*Persealingue* (Ruiz & Pav.) Nees	Magnoliophyta	Lauraceae	Terricolous	Tree	TE - ME	NA	1	1
42	*Peumusboldus* Molina	Magnoliophyta	Monimiaceae	Terricolous	Tree	ME	EN	1	1
43	*Pinusradiata* D. Don	Pinophyta	Pinaceae	Terricolous	Tree	TE - ME	IN	1	1
44	*Proustiapyrifolia* DC.	Magnoliophyta	Asteraceae	Liana	Shrub	ME	EN	1	1
45	*Raukauavaldiviensis* (Gay) Frodin	Magnoliophyta	Araliaceae	Liana	Shrub	TE	EN	1	1
46	*Sarmientascandens* (J.D. Brandis ex Molina) Pers.	Magnoliophyta	Gesneriaceae	Epiphyte	Subshrub	TE	EN	1	1
47	*Synammiafeuillei* (Bertero) Copel.	Polypodiopsida	Polypodiaceae	Epiphyte	Herb	TE - ME	NA	1	1
48	*Tropaeolumciliatum* Ruiz & Pav.	Magnoliophyta	Tropaeolaceae	Vine	Herb	TE - ME	EN	1	1
49	*Viciavicina* Clos	Magnoliophyta	Fabaceae	Vine	Herb	TE - ME	EN	1	1
